# 1-Year Fixed-Regimen Bevacizumab Treatment in DME-Vascular Network Image Analysis in Optical Coherence Tomography Angiography Study

**DOI:** 10.3390/jcm11082125

**Published:** 2022-04-11

**Authors:** Magdalena Hunt, Adam Wylęgała, Edward Wylęgała, Sławomir Teper

**Affiliations:** 1 Department of Ophthalmology, Faculty of Medical Sciences in Zabrze, Medical University of Silesia, 40-760 Katowice, Poland; wylegala@gmail.com (E.W.); slawomir.teper@gmail.com (S.T.); 2 Department of Ophthalmology, District Railway Hospital in Katowice, 40-760 Katowice, Poland; 3 Health Promotion and Obesity Management Unit, Department of Pathophysiology, Faculty of Medical Sciences in Katowice, Medical University of Silesia, 40-760 Katowice, Poland; adam.wylegala@gmail.com

**Keywords:** diabetic macular edema, AngioTool software, diabetic retinopathy

## Abstract

Purpose: To evaluate the effectiveness of intravitreal bevacizumab treatment in patients with diabetic macular edema (DME) by assessing retinal changes using AngioTool software (version 0.6a(02.18.14), National Cancer Institute, Bethesda, Maryland). Methods: A total of 27 eyes in patients with treatment-naïve DME were included in this prospective study. OCT-A images with a scan area of 6 × 6 mm were obtained. The DME patients with a central macular thickness (CMT) of ≥300 µm received nine bevacizumab injections within 12 months. The demographic, systemic, and ocular parameters, including the best-corrected visual acuity (BCVA) and CMT, were assessed. Explant area, vessels area, vessels percentage area, total number of junctions, total vessels length, average vessels length, the total number of endpoints, and mean lacunarity in the superficial capillary plexus (SCP) were calculated by using AngioTool software. Results: Twenty-nine eyes of DME patients were subjected to the final analysis. Bevacizumab treatment reduced CMT from 401.84 ± 84.54 µm to 328.93 ± 87.17 µm and improved BCVA from 65.18 ± 8.21 at baseline to 72.63 ± 7.43 letters among participants of the study. The anti-VEGF therapy showed no statistically significant changes in parameters calculated by AngioTool software in the study group of patients. Conclusion: The fixed-regimen intravitreal bevacizumab therapy was effective in treating DME. AngioTool software is an additional tool that could be used to assess vascular networks. However, the use of OCTA is unlikely to alter DME treatment regimens significantly or to find significant predictors. Perhaps using wide-angle devices or software will give a complete picture of the disease and prove to be more helpful.

## 1. Introduction

One of the common microvascular complications of both types (type 1 or type 2) of diabetes mellitus is diabetic retinopathy (DR) [1,2,3]. It is a leading cause of vision impairment that is observed worldwide, particularly among working-age adults in developed countries and the elderly [2,4,5]. Approximately 429 million people suffering from diabetes mellitus and more than 190 million with diabetic retinopathy are estimated to be affected by 2030, and the numbers are expected to grow due to lifestyle, obesity, and improved detection of the disease [4,6,7].

DR is categorized into two main types: nonproliferative DR and proliferative DR. Its severity corresponds with the dysregulation of the retinal vasculature. Early pathologic signs of DR are microaneurysms (MAs), expressed as capillaries dilatations primarily located in the central retina, especially in the deep capillary plexus (DCP). The development of MAs is a result of pericyte loss, the thickness of the basement membrane, and capillary endothelial cell dysfunction [6], whereas diabetic macular edema, the leading cause of vision impairment in patients with diabetes mellitus type 2, is characterized by the exudation from MAs and retinal capillary loss with high concentrations of cytokines. Moreover, the number of MAs, enlargement of the foveal avascular zone (FAZ), and presence of DME are necessary for diagnosis and staging of DR [1,6]. The progression of DME can be prevented by early detection, regular follow-up, and appropriate treatment. Currently, DME treatment is mainly based on intravitreal anti-VEGF injections. One of the anti-VEGF inhibitors used off-label for therapy patients with DME is bevacizumab [8,9].

Optical coherence tomography angiography (OCT-A) is currently the more often used examination for diagnostic and monitoring of DR. It is a noninvasive tool for mapping the movement of erythrocytes over time by comparing repeated B-scans that visualize each retinal vascular layer, especially superficial capillary plexus (SCP) and deep capillary plexus (DCP), without dye injection [1,2]. Studies have demonstrated microvascular changes detected on OCT-A, such as enlargement of the foveal avascular zone (FAZ), capillary nonperfusion area, and more MAs in DCP than in SCP in patients with diabetic retinopathy compared with healthy controls [2]. Despite many advantages of OCT-A, motion (connected with breathing, tremor, and pulsation) or doubling artifacts are frequent in the deeper layers due to shadows caused by moving blood cells in the overlying retinal vessels [1,10]. However, OCT-A quality could also be limited by massive intraretinal hemorrhages, exudation, or diffuse edemas, thus making OCT-A evaluation challenging.

AngioTool is one of the validated, publicly available software that could be used for calculating various morphological and spatial features of the vascular network [11,12,13]. AngioTool software has more functional capabilities available to the user, compared with built-in software, allowing more detailed evaluation of the vascular networks. For example, it provides a measure of the following features: total area covered by vessels within the complex that corresponds to the area of OCT-A flow information (vessel area); total size of the vascular complex (explant area); the sum of all vessels defined as the distance between two junctions or endpoints (total vessel length); total number of junctions; index for vascular structural non-uniformity (lacunarity) where higher values concern more heterogenous vasculatures [12,14,15,16,17].

The aim of this study was to assess the retinal vascular parameters measured with AngioTool in patients with DME treated with bevacizumab.

## 2. Materials and Methods

This prospective study was approved by the Ethics Committee of the Medical University of Silesia (KNW/0022/KB1/126/I/18/19). The study was conducted in accordance with the 1964 Declaration of Helsinki. Patients to this study were recruited from the ophthalmological outpatient clinic of the Clinical Department of Ophthalmology at the Faculty of Medical Sciences in the Medical University of Silesia throughout 2018–2020. Written informed consent was obtained from all the participants enrolled in the study.

Inclusion criteria for the study were as follows: (1) patients over 18 years old with type 1 or type 2 diabetes mellitus, (2) nonproliferative diabetic retinopathy, (3) diagnosis of DME with a central macular thickness (CMT) of ≥300 µm, (4) intravitreal treatment-naïve patients, and (5) best-corrected visual acuity (BCVA) of 24–78 ETDRS (Early Treatment Diabetic Retinopathy Study) letters. In addition, exclusion criteria were as follows: (1) history of any retinal surgery; (2) any previous intraocular treatment such as intravitreal injections of anti-VEGF agents/steroids or retinal photocoagulation; (3) media opacity disabling to assess the fundus of the eye (e.g., cataract, cornea abnormalities); (4) diagnosis of glaucoma; (5) presence of epiretinal membrane, vitreoretinal traction in the macula, or other types of maculopathy unrelated to diabetes mellitus (e.g., age-related macular degeneration); (6) unwilling to cooperate with OCT-A imaging, and (7) proliferative DR.

All the subjects were initially interviewed and examined during the routine visit. The following data were recorded: age, sex, height, weight, concomitant medications and duration of diabetes mellitus, concomitant systemic diseases (e.g., hypertension, history of heart incidents and stroke, chronic kidney disease), and serum level of glycated hemoglobin (HbA1c).

The participants of the study were treated with intravitreal injections of bevacizumab. Anti-VEGF inhibitors were performed by an ophthalmologist in the ophthalmological outpatient clinic of the Clinical Department of Ophthalmology at the Faculty of Medical Sciences in the Medical University of Silesia. Nine injections of 1.25 mg/0.05 mL bevacizumab (Avastin) were administered over 12 months in each studied eye. The first five injections were applied monthly, and the next four injections were given every two months.

The study patients had performed BCVA test, slit-lamp examination, OCT, and OCT-A before every injection. A swept-source OCT system (DRI OCT Triton; Topcon, Inc., Tokyo, Japan) with a wavelength of 1050 nm at a speed of 100,000 A-scans per second (each 512 × 512 mm) was used in OCT-A scans [18,19]. Two OCT-A scans centered on the fovea were performed with an area of 6 × 6 mm at baseline (up to 4 weeks before the first injection), every visit up to 4 h before intravitreal bevacizumab injection, and up to 4 weeks after the last bevacizumab injection. First, good quality OCT-A scans of individual segmented layers automatically—SCP and DCP—were assessed by the built-in IMAGEnet6 software (version 1.26.16898) [18,19]. Then, the SCP images of area 6 × 6 mm were analyzed by using AngioTool (version 0.6a (02.18.14)). Finally, two separate readers analyzed all the images.

Quantitative and qualitative analyses were made of 6 × 6 mm OCT-A scans. The AngioTool software was used each time to measure the vessel density of the OCT-A images as a validated tool for calculating vascular networks [11]. On opening images from AngioTool software, the same preset parameters, such as vessel density and intensity, were performed each time for every lesion, and all the results were compared [12]. The software skeletonizes and analyzes the area of interest [14]. Once the analysis is completed, the program extracts the results in a working spreadsheet. The results are extracted in a working spreadsheet each time after the completed analysis [17]. The following features as explant area, vessels area, vessels percentage area, total number of junctions, total vessels length, average vessels length, the total number of endpoints, and mean lacunarity of 6 × 6 mm area of OCT-A scans of the SCP were analyzed by the AngioTool software. Vessel area is defined as the total area covered by vessels within the complex that corresponds to the area of OCT-A flow information; explant area is the total size of the vascular complex; total vessel length is the sum of all vessels defined as the distance between two junctions or endpoints; the total number of junctions; lacunarity is the index for vascular structural non-uniformity, where higher values concern more heterogenous vasculatures but lower values equal homogeneous vasculatures [12,14,15,16,17]. Motion or doubling artifacts, blurry images, quality scores lower than 40, or poor centration are exclusion criteria of the analysis.

Diabetic macular edema (DME) in our study was defined as a central macular thick-ness (CMT) of ≥300 µm, and only one eye of each patient was assessed in the study. If both eyes were affected, only one eye was randomly selected to be evaluated.

## 3. Results

A total of 27 eyes of 27 subjects were included in the study. The mean age of the participants was 68.00 ± 8.60. An average participant had DM for 12.69 ± 8.01 years. Among the study participants, 11 men were included (41%). Table 1 presents demographic information and corneal parameters of all subjects. Most eyes included in the study were phakic (66%), and the mean axial length was 23.29 ± 0.74 mm. Most participants were suffering from hypertension (89%), while the complications of DMt2 were not so commonly found in our study group, with chronic kidney disease and brain stroke found in 11% and 7%, respectively. DMt2 was primarily treated with tablets (59%), insulin-only therapy was less common (22%), while combined therapy was present in (26%).

The anti-VEGF therapy showed no statistically significant changes in explant area, *p* = 0.7, vessel area *p* = 0.6, vessel percentage area *p* = 0.4, the total number of junctions *p* = 0.4, total vessel length *p* = 0.3, average vessels length *p* = 0.6, the total number of endpoints *p* = 0.7, and mean lacunarity *p* = 0.7 within the tested period (Table 2).

**Figure 1:**
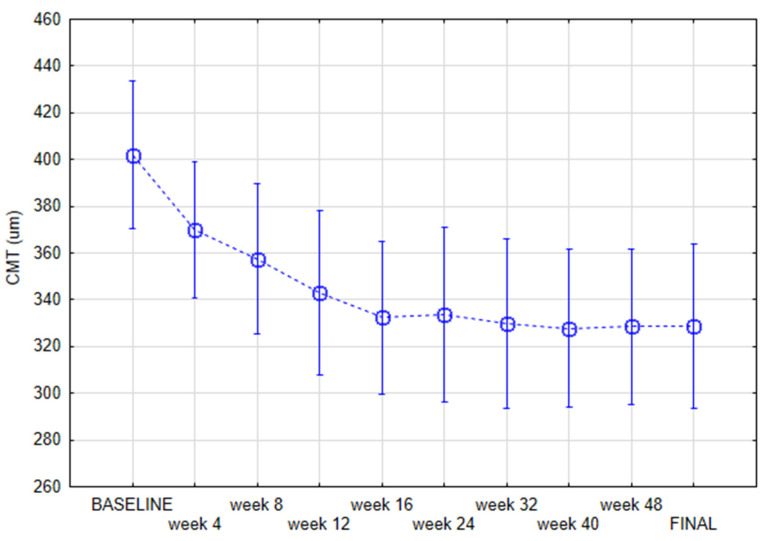
Central macular thickness (CMT) during the case of the study measured with repeated measures ANOVA current effect: F(9, 225 = 9.7280, *p* < 0.001).

We found using repeated measures ANOVA that CMT value decreased during the course of the therapy significantly from 401.84 ± 84.54 at baseline to 328.93 ± 87.17 µm at the final visit (*p* < 0.001) (Figure 1).

**Figure 2:**
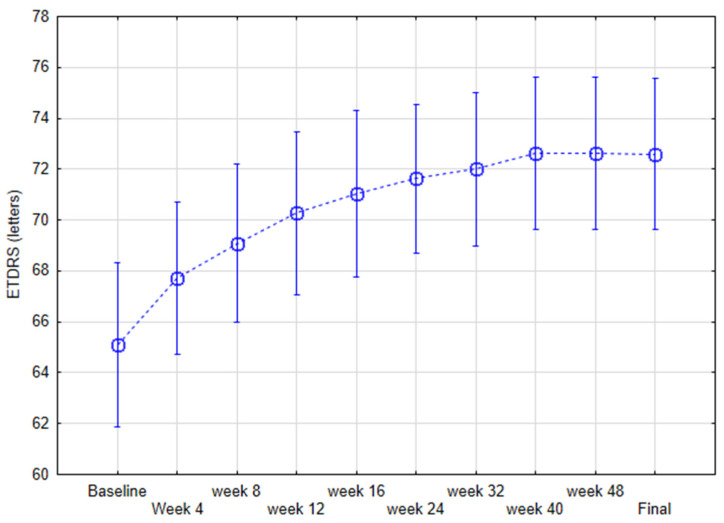
Early Treatment Diabetic Retinopathy Study (ETDRS) scores during the course of the study measured with repeated measures ANOVA current effect: F(9, 225 = 39.334, *p* < 0.001).

Anti-VEGF therapy resulted in statistically significant difference in the mean ETDRS scores from 65.18 ± 8.21 at baseline to 72.63 ± 7.43 letters (*p* < 0.001) (Figure 2).

**Figure 3:**
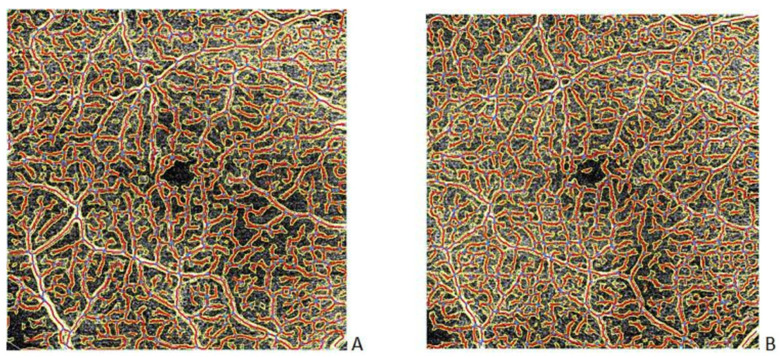
An example of vascular network of the SCP before (**A**) and after (**B**) treatment of bevacizumab intravitreal injections assessed with AngioTool software.

OCT-A scans of 6 × 6x mm area from the SCP were assessed by AngioTool software each time before (Figure 3A), during, and after treatment (Figure 3B) of bevacizumab intravitreal injections.

## 4. Discussion

Our study demonstrated that 12-month therapy of intravitreal bevacizumab injections for diabetic macular edema was effective. SS-OCT and OCT-A were used to assess the effectiveness of the anti-VEGF treatment. This study showed that fixed-protocol of intravitreal bevacizumab treatment effectively improves BCVA and CMT reduction.

Anti-VEGF inhibitors are often used to treat diabetic macular edema caused by in-creased vascular permeability in the retina. Bevacizumab is a human monoclonal anti-VEGF antibody whose effectiveness on DME treatment has been proven in many studies. The positive effects of bevacizumab intravitreal injections included—improved visual acuity and reduced central macular thickness in DME patients [20]. A multicenter randomized study conducted by the Diabetic Retinopathy Clinical Research Network compared three usually applied intravitreal anti-VEGF inhibitors—aflibercept, ranibizumab, and bevacizumab—for DME therapy. An average improvement in BCVA letter score was 12.8 letters with aflibercept, 12.3 letters with ranibizumab, and 10.0 letters with bevacizumab from baseline to the 2-year visit. Moreover, reduction in CMT from baseline to the 2-year visit was on average 171 ± 141 microns with aflibercept, 149 ± 141 microns with ranibizumab, and 126 ± 143 microns with bevacizumab group. During this 2-year study, the median numbers of intravitreal injections were 15 (aflibercept), 15 (ranibizumab), and 16 (bevacizumab) [9]. In addition, 5-year follow-up showed that the average BCVA letter score improved from baseline to 5 years by 8.0 letters in the aflibercept group, 7.6 letters in the ranibizumab group, and 6.6 letters in the bevacizumab group. Furthermore, mean 5-year CMT decreased from baseline by 161 m with aflibercept, 150 m with ranibizumab, and 150 m with bevacizumab group [21]. In a study by Ekinci et al., they compared bevacizumab and ranibizumab intravitreal treatment on visual acuity and CMT at 12-month follow-up. The mean decrease in CMT was from 483.8 ± 126 m at baseline to 342.3 ± 121 m at the 12th month after treatment, with the mean number of 5.1 ± 0.74 injections within bevacizumab group of fifty participants. In comparison, improvement in BCVA was on average from 0.22 ± 0.11 to 0.38 ± 0.12, finally measured on Snellen chart [20]. On the other hand, Nepomuceno et al. observed during 48 weeks follow-up a significant improvement in mean BCVA according to logMAR and significant reduction in central subfield thickness compared with baseline in both bevacizumab and ranibizumab groups. However, they reported that the mean number of injections was higher in the bevacizumab group (9.84 ± 0.55 injections) than in the ranibizumab group (7.67 ± 0.60 injections) [22]. Our results show a similar trend concerning BCVA letter score and CMT as these findings, while the main differences between studies resulted from the duration of the study, amount of participants, type of anti-VEGF medication, and the number of injections applied.

Our study focused on SCP changes among treatment-naïve DME patients during 12 months of intravitreal bevacizumab therapy. Several OCT-A-based studies have compared different parameters. It is difficult to obtain reliable vascular measurements from OCT-A using built-in tools in many devices. For this reason, other methods of quantifying angio-OCT data are chosen. One of them is AngioTool software, where OCT-A images of a 6 × 6 area were also analyzed. Several parameters were assessed, such as an area of segmented vessels (vessels area), the overall size of the vascular network (explant area), vessels percentage area, total and average vessel length, the total number of endpoints or junctions, and lacunarity, which is an index of vascular non-uniformity [12,16]. More heterogeneous vessel organization is associated with a higher lacunarity, while lower lacunarity shows the vascular structure as being more uniform and homogeneous [12,14,15,16,17]. According to a Liu et al. study, identification of capillaries is sufficient only in live retinal vessels in contrast with the postmortem vascular network of mice retinas [23]. Reliable and reproducible results are only possible within the superficial capillary plexus—SCP. Sun et al. in their study showed associations of AngioTool-assessed vessel density in 3 × 3, 6 × 6, and 8 × 8 mm OCT-A among 247 cardiovascular patients. They demonstrated that increasing age, hypertension, dyslipidemia, coronary artery disease, aspirin, second antiplatelet, ACEI/ARBs, and statin use were connected with decreased average vessel length in 3 × 3 mm OCT-A scans, while in 6 × 6 mm scans the only statistically essential connection was increasing age, and in addition in 8 × 8 mm OCT-A scans there were no statistically significant connections. Moreover, vessel perfusion, as well as vessel length and junction density, was associated with higher blood glucose and glycated hemoglobin in 3 × 3 mm OCT-A angiograms but not in larger scans (as 6 × 6 mm or 8 × 8 mm) [13]. Recent studies conducted by Told et al. and Schranz et al. among a small group of treatment-naive nAMD patients showed a solid response to anti-VEGF treatment. Both groups of researchers proved that vessel area, total vessel length, and the total number of junctions responded to anti-VEGF inhibitors with a decrease within 7 to 14 days after the first injection, followed by an increase until one month after treatment. However, they also noticed that no statistical significance occurred after second and third anti-VEGF injection in these parameters [14,15]. In addition, Schranz et al. could not find a response pattern for aflibercept treatment associated with average vessel length, vessel percentage area, total number of vessel end points, and mean lacunarity [15]. If the parameters were significantly various in particular groups of patients differently responding to treatment, they could be used as predictors. According to a Zeydanli et al. study, forty-one eyes of patients with nAMD, who were treated with anti-VEGF injections, were divided into groups of “good responders” (22 eyes) and “poor responders” (19 eyes) based on morphological and functional aspects, with a median follow-up of over 40 months. Significantly higher lacunarity values were noticed in the good responders group compared with the group of poor responders, and conversely, lower lacunarity values were observed among poor responders rather than the group of good responders. Moreover, statistically insignificant parameters such as junction density were insensibly higher in the poor responders than in the good responders [12]. However, our study did not demonstrate a statistically significant association in terms of parameters calculated by AngioTool software before and after treatment. It is difficult to obtain reliable vascular measurements with OCT-A using built-in tools in many devices [24,25]. For this reason, other methods of quantifying angio-OCT data are available. One of them is AngioTool software. Using this method, it was possible to provide reliable and reproducible results from a superficial plexus [11]. What is more, it is a helpful tool for calculating and examining the vascular network in OCT-A images. Nevertheless, it has not proven to be helpful for monitoring changes during DME treatment. On the other hand, a small number of participants is the limitation of that study, and further investigation is needed to look for more subtle factors.

## 5. Conclusions

In conclusion, intravitreal bevacizumab therapy is effective in DME treatment. AngioTool software is an additional tool that could be used for the assessment of a vascular network. OCTA is unlikely to alter DME treatment regimens significantly or to find significant predictors. Perhaps using wide-angle devices or software development will give a complete picture of the disease and prove to be more useful.

## Figures and Tables

**Table 1:** Qualitative and quantitative variables of patients with diabetic macular edema (DME) qualified for the study described as unsatisfactory.

Variable	Mean	Minimum	Maximum	SD.
Age	68.00	48.00	84.00	8.60
Body mass index	26.71	19.38	39.79	4.33
Time of diabetes	12.69	4.00	31.00	8.01
HbA1c%	7.36	5.70	9.40	1.09
Axial length	23.29	21.53	24.96	0.74
Spherical equivalent	0.81	−1.50	2.50	1.14
Cylindrical value	−0.42	−1.50	0.00	0.50
IOP	15.79	12.00	21.00	2.04
			number	%
Gender	Male		11	41
	Female		16	59
Chronic kidney disease			3	11
Hypertension			18	67
Myocardial ischemic disease			6	22
Myocardial infarction			4	15
Brain stroke			2	7
Lens status (pseudophakia)			10	34
Tablets			16	59
Insulin			6	22
Combined therapy			7	26

SD = standard deviation.

**Table 2:** OCT angiography parameters at baseline and at the final visit were measured with repeated measures ANOVA.

Variable		Mean	SD	Mean	SD	Repeated Measures ANOVA *p*-Value
Explant area	15	260,506.1	1058.212	282,082.8	73,277.40	0.7
Vessels area	15	106,547.1	7182.383	118,541.1	29,471.49	0.6
Vessels percentage area	15	40.9	2.716	42.1	1.73	0.4
Total number of junctions	15	556.0	61.582	594.8	153.88	0.4
Total vessels length	15	16,906.3	934.952	18,537.3	4783.51	0.3
Average vessels length	15	214.2	61.409	224.9	46.03	0.6
Total number of end points	15	457.3	43.225	469.4	112.43	0.7
Mean lacunarity	15	0.05	0.01	0.05	0.01	0.7

## Data Availability

Data available on request due to restrictions, e.g., privacy or ethical.
